# Efficiency and effectiveness evaluation of an automated multi-country patient count cohort system

**DOI:** 10.1186/s12874-015-0035-9

**Published:** 2015-05-01

**Authors:** Iñaki Soto-Rey, Benjamin Trinczek, Yannick Girardeau, Eric Zapletal, Nadir Ammour, Justin Doods, Martin Dugas, Fleur Fritz

**Affiliations:** Institute of Medical Informatics, University of Münster, Albert-Schweitzer-Campus 1/A11, D-48149 Münster, Germany; Département d’Informatique Hospitalière, AP-HP, Hôpital Européen Georges Pompidou, 75015 Paris, France; Centre de Recherche des Cordeliers, Sorbonne Universités, UPMC Univ Paris 06, UMR_S 1138, F-75006 Paris, France; Sanofi-Aventis R&D, 1 avenue Pierre Brossolette, F-91380 Chilly-Mazarin, France

**Keywords:** Clinical trials as topic, Evaluation studies, Feasibility studies, Electronic health records

## Abstract

**Background:**

With the increase of clinical trial costs during the last decades, the design of feasibility studies has become an essential process to reduce avoidable and costly protocol amendments. This design includes timelines, targeted sites and budget, together with a list of eligibility criteria that potential participants need to match.

The present work was designed to assess the value of obtaining potential study participant counts using an automated patient count cohort system for large multi-country and multi-site trials: the Electronic Health Records for Clinical Research (EHR4CR) system.

**Methods:**

The evaluation focuses on the accuracy of the patient counts and the time invested to obtain these using the EHR4CR platform compared to the current questionnaire based process. This evaluation will assess the patient counts from ten clinical trials at two different sites. In order to assess the accuracy of the results, the numbers obtained following the two processes need to be compared to a baseline number, the “alloyed” gold standard, which was produced by a manual check of patient records.

**Results:**

The patient counts obtained using the EHR4CR system were in three evaluated trials more accurate than the ones obtained following the current process whereas in six other trials the current process counts were more accurate. In two of the trials both of the processes had counts within the gold standard’s confidence interval.

In terms of efficiency the EHR4CR protocol feasibility system proved to save approximately seven calendar days in the process of obtaining patient counts compared to the current manual process.

**Conclusions:**

At the current stage, electronic health record data sources need to be enhanced with better structured data so that these can be re-used for research purposes. With this kind of data, systems such as the EHR4CR are able to provide accurate objective patient counts in a more efficient way than the current methods.

Additional research using both structured and unstructured data search technology is needed to assess the value of unstructured data and to compare the amount of efforts needed for data preparation.

**Electronic supplementary material:**

The online version of this article (doi:10.1186/s12874-015-0035-9) contains supplementary material, which is available to authorized users.

## Background

### Scientific background

Clinical Trials (CTs) are essential for the improvement and development of preventive and therapeutic strategies, epidemiology and healthcare. The complexity and cost of carrying out CTs have increased over the last few decades [1]. Oftentimes they are delayed or even cancelled, which results in a tremendous loss for the institutions that fund them [2].

As a strategy to avoid such loss, it has been established that every CT starts with a feasibility study, defined by Arain as a “piece of research done before a main study, […] used to estimate important parameters that are needed to design the main study” [3]. These parameters are, besides others, timelines, targets and costs of performing a CT in determined geographical regions. To tackle the key issue of low recruitment rates during the conduction of CTs, feasibility studies also include a list of eligibility criteria (EC) that potential study participants need to match. The study design process starts with a series of meetings, through which the feasibility study team decides the main objectives of the study and produces an initial study draft, called protocol synopsis. Secondly, one or several country feasibility managers evaluate the feasibility of the trial in certain countries. This process requires basic feasibility data such as patient counts that would match the trial’s EC. Those counts are usually obtained by consulting clinicians at different sites within the countries - a costly and inefficient process [4]. In the next step of the protocol design, site feasibility managers ask potential study sites about their commitment regarding the number of patients they can enrol and other relevant information for the trial such as technological and human resources. Based on all this information, the feasibility study team decides on the final list of sites and the study protocol, which both get documented in the final study design [5].

Modifications of the study protocol lead to the repetition of: cohort definition by criteria, approaching the country feasibility managers and sites, estimations collection and finally comparison of these estimations to a recruitment goal and necessary cohort size respectively. All these repetitions result in cumbersome, slow and costly work.

There is general consensus that a well-defined study protocol leads to fewer protocol amendments, and thus a decrease in the final trial cost [6]. Eventually, better, faster and less costly feasibility studies lead to a better success rate of clinical trials [7]. Several experts agree on the need for improvement of the protocol feasibility phase, and even categorize it as one with the greatest potential for improvement [8].

There is a growing awareness of the importance of electronic health records (EHRs) for the improvement of healthcare [9] and clinical research [10].

With the purpose of reusing EHR data from different countries to support clinical trials [11], the Innovative Medicine Initiative (IMI) [12] launched one of its biggest initiatives in 2010: the Electronic Health Records for Clinical Research (EHR4CR) project [13], in which this research was focused.

### Rationale for the study

The EHR4CR project includes four scenarios that cover the clinical trial steps of: (1) clinical protocol feasibility (PF), (2) patient identification and recruitment, (3) clinical trial execution and (4) adverse event reporting. The main objectives of the first phase of the project are to support the design of study protocols and to improve the country feasibility step of PF. The EHR4CR PF system supports these processes by providing patient counts for certain user-selected EC from several sites across different countries. Thus, clinicians do not need to be approached when seeking for eligible patient counts and objective results can be obtained.

Several internal tests proved the reliability of the platform and the effectiveness of the algorithm that calculates the eligible patients within the EHR4CR local data-warehouses, but all these experiments were based on test data and an evaluation of the platform performance in a real scenario was still needed.

### Objectives of the study

To test the reliability of the EHR4CR PF system in a real-case scenario, the objective of this work is to evaluate the effectiveness, defined by the International Organization for Standardization as: accuracy and completeness with which users achieve specified goals; and the efficiency: resources expended in relation to the accuracy and completeness with which users achieve goals [14]; in the processes of obtaining patient counts that match certain EC. A comparison was made between the current manual processes to obtain these counts and the counts obtained using an automated patient count cohort system such as the EHR4CR PF system.

This research was approved by the internal EHR4CR scientific committee, the Münster ethics committee and the data protection managers and clinic directors of the sites involved in the evaluation.

### Study context

Two EHR4CR sites participated in this evaluation: the Assistance Publique-Hôpitaux de Paris in France (AP-HP) and the University Hospital Münster in Germany (UKM), involving two clinics from the AP-HP (Departments of Urology and Pneumology) and 7 from the UKM (Departments of Medicine A, B, C, D, Urology, Neurology and Gynaecology).

### System details and systems in use: The EHR4CR system

The EHR4CR PF technical platform is based on a complex architecture [15], with an algorithm that transforms graphically user-created sets of rules into machine readable language. A web based information system has been developed to facilitate the creation and management of protocol feasibility studies, each of them containing one or several protocol feasibility queries. This EHR4CR PF system contains a so called “query builder”, which uses specific EHR4CR terminology services [16] for the selection of EC. The query builder also contains as well temporal constrains (e.g. X before/after Y) and all the necessary Boolean logic to build PF queries. These global queries are sent though certified secured web-channels to the user selected endpoints located at the hospital sites. The queries are then executed at each selected site and the patient counts matching the EC are sent back to the EHR4CR PF system where they can be visualized.

The EHR4CR PF system ensures the anonymity of the data displaying only patient counts higher than five and fuzzing the counts when the result is lower than five. Several of the EHR4CR data providers also shifted the dates of the data elements within their EHR4CR data warehouses between zero and 365 days.

### Selection of studies

At the beginning of the year 2011, all ten European Federation of Pharmaceutical Industries and Associations (EFPIA) companies participating in the project were asked to deliver a list of recently completed (within the last two years) or ongoing studies (with completed feasibility phase) running at the participating sites.

From the initial list of 267 trials, ten studies were selected based on criteria so that a) each EFPIA company and b) each data provider site participating in the EHR4CR project were represented at least once. Studies running at several sites were prioritized from those running at a fewer sites.

Feasibility experts from the collaborating EFPIA partners simplified the free text EC of these studies and extracted ‘non-ambiguous data elements’. Since this work was focused on PF, criteria that were not essential for feasibility were removed (e.g. in the case of the NCT01018173 trial, 26 criteria were extracted after the simplification, but for feasibility only 5 were relevant). Table 1 summarizes the ten studies and shows how many criteria were selected as feasibility EC.

**Table 1:** **List of selected studies**

**Title**	**EFPIA partner**	**Disease category**	**Original number of EC**	**Number of feasibility criteria**
Once – Daily Oral Direct Factor Xa Inhibitor Rivaroxaban In The Long-Term Prevention Of Recurrent Symptomatic Venous Thromboembolism In Patients With Symptomatic Deep-Vein Thrombosis Or Pulmonary Embolism. The Einstein-Extension Study	Bayer	Cardiovascular- PE/DVT	6	6
E.V.O.L.V.E. Trial™: Evaluation Of Cinacalcet HCl Therapy to Lower CardioVascular Events	Amgen	Cardiovascular	3	3
Safinamide in Idiopathic Parkinson’s Disease (IPD) With Motor Fluctuations, as add-on to Levodopa (SETTLE)	Merck	Nervous system disorders- Parkinson	19	5
Six Months Efficacy and Safety of Aliskiren Therapy on Top of Standard Therapy, on Morbidity and Mortality in Patients With Acute Decompensated Heart Failure	Novartis	Cardiovascular	22	16
Abiraterone Acetate in Castration-Resistant Prostate Cancer Previously Treated With Docetaxel-Based Chemotherapy	Janssen	Oncology	11	11
A Phase III Trial of ZD4054 (Zibotentan) (Endothelin A Antagonist) in Non-metastatic Hormone Resistant Prostate Cancer (ENTHUSE M0)	Astra-Zeneca	Oncology	30	3
GLP-1 Agonist AVE0010 in Patients With Type 2 Diabetes for Glycemic Control and Safety Evaluation, on Top of Basal Insulin	Sanofi	Diabetes	20	20
A Study of Taspoglutide in Patients With Inadequately Controlled Diabetes Mellitus Type 2 and Cardiovascular Disease	Roche	Cardiovascular + Diabetes	25	3
A Study in Participants With Type 2 Diabetes Mellitus	Lilly	Diabetes	16	16
ALTTO (Adjuvant Lapatinib And/Or Trastuzumab Treatment Optimisation) Study; BIG 2-06/N063D	GSK	Oncology	26	26

Details of the selected studies (name, company, disease areas) together with the original number of EC vs the reduced number of feasibility EC.

The EC data elements were then used to identify the corresponding data in the EHRs and mappings from local to central terminology codes were created. The clinical data warehouses (CDWs) at each site were filled with the mapped EHR data; so that queries generated using the EHR4CR PF system could retrieve valid patient counts from the sites. This process is also known as the Extraction, Transformation and Load of data (ETL).

## Methods

The evaluation process of the EHR4CR PF scenario started in May 2013 with the target decision of the evaluation. The studies used for the evaluation were the same ten trials used for building the PF scenario and the data utilized were from 2012.

### Effectiveness

The first objective of this evaluation was to evaluate the accuracy of the patient counts provided by the EHR4CR system and the current manual process. This was achieved by comparing these two values to a gold standard (defined below).The accuracy of the two processes was assessed by analysing whether the EHR4CR system produces closer counts to the gold standard than the current manual process (hypothesis) or vice versa. Therefore, three different values needed to be obtained for the effectiveness evaluation: the patient counts obtained following the current manual process, the ones obtained using the EHR4CR PF system and those belonging to the gold standard.

### Simulation of the current manual process

For each of the selected studies, a questionnaire was sent to a clinician of the participating sites in order to collect their estimation of the number of patients that would be eligible to participate in the respective study per year at their clinic or department. EFPIA members involved in the evaluation made necessary arrangements with the sites to introduce the subject and seek their participation. The questionnaire contained the estimation of matching patients and two additional questions about the common PF process and current PF issues that were used for the interpretation of the results in the discussion. The procedure to answer the questionnaire should be the same as the one the clinicians normally follow in order to answer to clinical trial feasibility questionnaires. An example of the questionnaire can be found in the supplement [see Additional file 1].

If the evaluated site was not part of the original study, the site itself was responsible for obtaining the estimations. The method followed was to contact the clinic directors and forward them the questionnaire so they could find the right person to answer it and return the results.

### New process: the EHR4CR PF system

The study population used for the evaluation consisted of all patients who visited the evaluated clinics during the year 2012. Seven different EHR4CR databases were created at UKM and two at AP-HP corresponding to the number of clinics evaluated at each site.

A medical expert with basic knowledge of the EHR4CR PF system created the studies using the EHR4CR query builder. The queries were then executed and the results stored. The complete set of queries can be found in the supplement [see Additional file 2].

The temporal constraints of the EHR4CR queries were adapted according to the date shift of the EHR4CR data elements: As the date of the elements is shifted in some of the sites between zero and 365 days, two criteria related to each other with both a temporal constraint shorter than 365 days could be biased.

### Chart Review - “Alloyed” gold standard

Gold standard [17] was defined as the result from a manual check of patient records. Due to the impracticability to review several thousands of patient records, an alloyed gold standard [18] was used instead, recurring to Wilson Score Confidence Interval for Binomial Proportion (CI = WILSON) [19] to build it.

The two-sided *100(1-α)%* confidence interval for *ρ* is:

$$ \frac{X+\frac{z_{1-\frac{\alpha }{2}}^2}{2}}{N+{z}_{1-\frac{\alpha }{2}}^2}\pm \frac{z_{1-\frac{\alpha }{2}}{N}^{\frac{1}{2}}}{N+{z}_{1-\frac{\alpha }{2}}^2}{\left(\widehat{\rho}\left(1-\widehat{\rho}\right)+\frac{z_{1-\frac{\alpha }{2}}^2}{4N}\right)}^{\frac{1}{2}} $$

So the half-width for the two-sided *100(1-α)%* confidence interval is:

$$ half\_ width=\frac{z_{1-\alpha /2}{N}^{\frac{1}{2}}}{N+{z}_{1-\alpha /2}^2}{\left(\widehat{\rho}\left(1-\widehat{\rho}\right)+\frac{z_{1-\alpha /2}^2}{4N}\right)}^{\frac{1}{2}} $$

Prob(Width) is calculated exactly by adding up the probabilities of observing each *X ϵ {1……N}* that produces a confidence interval whose half-width is at most a target value *h*:

$$ Prob(Width)={\displaystyle \sum_{i=0}^N}P\left(X=i\right){l}_{half\_ width < h} $$

According to this formula, checking at least 93 patients would be sufficient to then extrapolate and obtain a 95% of confidence. In some cases, more than one of the trials covered the same disease category, and we therefore used the same set of patient records for those studies. For example, if trial A and trial B are diabetes mellitus type II studies, the same set of patient records can be used to assess the eligibility of each patient for the two trials. The patient records were manually reviewed by medically qualified personnel from the evaluated sites.

After the initial review, the results were checked a second time and conflicting cases were discussed with the responsible clinicians, who decided the eligibility of the patient. Once all the results were obtained, the number of eligible patients from the seven (at UKM) and two (at AP-HP) manually checked 100-patients sets were extrapolated.

### Efficiency

The second objective of the evaluation was to estimate the time required in obtaining patient counts that match certain EC first following the current manual process and second using the EHR4CR PF system. Evaluating the efficiency of these processes does not require an “alloyed” gold standard in this context as the gold standard would be the faster process. In the first case, the value is obtained by measuring the time involved in preparing the questionnaire, sending it and obtaining the results. For the EHR4CR PF process, the time value is obtained by measuring the time invested in logging into the EHR4CR platform, creating the queries and obtaining the visualisation of the results. This value was obtained using the stopwatch method.

### 100 patient records test

The first results from the UKM site showed an unexpected high number of mismatches between the “alloyed” gold standard and the EHR4CR PF system. With the purpose of discovering the reason for this mismatch, an additional in-depth analysis of the cohorts was conducted at UKM. To retrieve the necessary information, a dedicated EHR4CR database with the 100 patient records manually checked for the NCT01018173 Roche trial was created. Each entry in the database was analysed separately to find the reasons for the differences between the PF system’s calculated numbers and the “alloyed” gold standard.

## Results

### Effectiveness

The first values required for the evaluation were the patient counts obtained by simulating the current process: the responses given from clinicians to the protocol feasibility questionnaire. A total number of nine clinicians (two at AP-HP and seven at UKM) were contacted: three of them (two at AP-HP, and one at UKM) by the corresponding EFPIA companies and the other six directly by the sites. All clinicians responded with estimated patient counts and feedback about the current PF process and issues. The estimations cover a range from ten to 340 potentially matching patients per year and clinic, based on the correspondent EC. The free text feedback from the clinicians can be found in the supplement [see Additional file 3]. Two of the clinicians stated that they contrast their opinion with health records in order to answer to feasibility questionnaires. Other three, that they base their responses on their own experience. The rest of the clinicians either did not answer to the question or the response was too ambiguous to consider it. The complete set of responses will be used for interpretation in the discussion.

The second value required was the result of the feasibility process using the EHR4CR PF system. All executed queries yielded in calculated patient counts, with results from zero to 695 matching patients. Table 2 lists the calculated patient counts for the queries executed at UKM. As it can be seen in Table 3, the results for AP-HP contain a higher number of patients matching the study criteria.

**Table 2:** **Results of the query execution using the EHR4CR PF system at UKM**

**Study** **EFPIA partner**	**C1**	**C2**	**C3**	**C4**	**C5**	**C6**	**C7**	**C8**	**C9**	**C10**	**C11**	**Eligible patients**	**Patients per clinic**
NCT00439725 Bayer	1411	30	0									0	1411
NCT00345839 Amgen	0	15	23	−7*								0	7439
NCT00627640 Merck	4331	128	28	<5	-<5*							0	5606
NCT00894387 Novartis	1884	412	0	884	0*	0*	−341*	0*	0*	−163*	0*	0	1887
NCT00638690 Janssen	1437	174	64	0	64	0*						0	1540
NCT00626548 AstraZeneca	1158	1437	174									174	1540
NCT00715624 Sanofi	573	18	−400*	−57*	0*							18	2575
NCT01018173 Roche	2570	573	44									566	2575
NCT01468987 Eli Lilly	573	775	0*	−8*	−53*	0*	−106*	−89*	−301*	11		8	2575

Columns C1 to C11 show the number of eligible patients for every single criterion, followed by the final number of eligible patients and the total number of patients who visited the respective clinics in the year 2012.

Results for the GSK- NCT00490139 trial are excluded due to formatting issues and can be seen in the Additional file 2.

*Exclusion criterion.

**Table 3:** **Results of the query execution using the EHR4CR PF system at AP-HP**

**Study EFPIA partner**	**C1**	**C2**	**C3**	**C4**	**C5**	**C6**	**Eligible patients**	**Patients per clinic**
NCT00439725 Bayer	4108	787	313				205	4116
NCT00638690 Janssen	8594	695	356	35	212	−24*	5	8626
NCT00626548 AstraZeneca	8594	6745	695				695	8626

Columns C1 to C6 show the number of eligible patients for every single criterion, followed by the final number of eligible patients and the total number of patients who visited the respective clinics in the year 2012.

*Exclusion criterion.

The results of the chart review for the evaluated studies at the two sites can be visualized in the Tables 4 and 5. Eligible patients were found in a range between zero and 34 patients out of the 100-patient records sets. These numbers were extrapolated using the ci-Wilson score resulting in a range between zero and 1035 eligible patients between the lowest and the highest means. The lower ci-Wilson bounds vary between zero and 655 patients and the upper bounds between 54 and 1709 patients.

**Table 4:** **Results of the chart review at UKM**

**Study EFPIA partner**	**Results chart review**	**Gold Standard-Mean [LB – HB]**	**Patients per clinic**
NCT00439725 Bayer	3	42 [14 – 120]	1411
NCT00345839 Amgen	1	74 [13 – 406]	7439
NCT00627640 Merck	2	112 [30 – 393]	5607
NCT00894387 Novartis	4	75 [29 – 186]	1885
NCT00638690 Janssen	2	31 [8 – 108]	1540
NCT00626548 AstraZeneca	14	216 [131 – 341]	1540
NCT00715624 Sanofi	0	0 [0 – 96]	2575
NCT01018173 Roche	34	876 [655 – 1126]	2575
NCT01468987 Eli Lilly	10	257 [142 – 449]	2575
NCT00490139 GSK	4	22 [8 – 54]	546

Matching patients for the 100 records manually checked at UKM, total number of patients per clinic and mean result of the extrapolation using the ci-Wilson score with a 95% of confidence together with the lower and upper bounds and the total number of patients who visited the clinic in the year 2012.

**Table 5:** **Results of the chart review at AP-HP**

**Study EFPIA partner**	**Results chart review**	**Gold Standard-Mean [LB – HB]**	**Patients per clinic**
NCT00439725 Bayer	12	494 [288 – 816]	4116
NCT00638690 Janssen	1	86 [15 – 470]	8626
NCT00626548 AstraZeneca	12	1035 [603 – 1709]	8626

Matching patients for the 100 records manually checked at AP-HP, total number of patients per clinic and mean result of the extrapolation using the ci-Wilson score with a 95% of confidence together with the lower and upper bounds and the total number of patients who visited the clinic in the year 2012.

The comparison between the three values: estimations from clinicians, results from the EHR4CR PF system and chart reviews (Tables 6 and 7), shows that the values from EHR4CR PF system were within the bounds of the confidence interval in three cases. In one of those three cases the estimation from the clinician was below the lower bound, in the other two cases the clinician estimations were as well within the confidence interval. In ten other cases, the EHR4CR PF system values were below the lower bound. In six out of these ten cases, the clinician estimations were within the confidence interval. Out of the four cases in which both values were under the lower bound of the “alloyed” gold standard, in two of them the response from the EHR4CR system was closer to the “alloyed” gold standard and in the other two the estimations from the clinicians were the closer ones. As a summary, the counts provided by the EHR4CR PF system were the more accurate ones in three out of the thirteen cases (two from AP-HP and one from UKM), whereas in six other cases, the more accurate counts were the clinician estimations (five from UKM and one from AP-HP). In two out of the thirteen cases both of the processes provided counts within the confidence interval of the “alloyed” gold standard.

**Table 6:** **Overall results at UKM**

**Study EFPIA partner**	**Results current process**	**Results EHR4CR PF system**	**Gold Standard-Mean [LB – HB]**	**Patients per clinic**
NCT00439725 Bayer	30	0	42 [14 – 120]	1411
NCT00345839 Amgen	200	0	74 [13 – 406]	7439
NCT00627640 Merck	300	0	112 [30 – 393]	5607
NCT00894387 Novartis	25	0	75 [29 – 186]	1885
NCT00638690 Janssen	50	0	31 [8 – 108]	1540
NCT00626548 AstraZeneca	200	174	216 [131 – 341]	1540
NCT00715624 Sanofi	12	18	0 [0 – 96]	2575
NCT01018173 Roche	340	566	876 [655 – 1126]	2575
NCT01468987 Eli Lilly	110	8	257 [142 – 449]	2575
NCT00490139 GSK	10	0	22 [8 – 54]	546

Overview of the results obtained by current and EHR4CR supported processes for each of the trials evaluated at UKM and comparison with the gold standard.

**Table 7:** **Overall results at AP-HP**

**Study EFPIA partner**	**Results current process**	**Results EHR4CR PF system**	**Gold Standard-Mean [LB – HB]**	**Patients per clinic**
NCT00439725 Bayer	20	205	494 [288 – 816]	4116
NCT00638690 Janssen	25	5	86 [15 – 470]	8626
NCT00626548 AstraZeneca	250	695	1035 [603 – 1709]	8626

Overview of the results obtained by current and EHR4CR supported processes for each of the trials evaluated at AP-HP and comparison with the gold standard.

### Efficiency

The creation, execution and visualization of a query using the EHR4CR PF system required between five and 25 minutes depending on the complexity of the query (five minutes for a query with three criteria and 25 with 26 criteria).

The time required to receive the response from the PIs varied depending on whether the questionnaire was sent by the EFPIA representative or directly by the site. In the former case, the response to the questionnaire was received in seven calendar days, whereas in the latter it required between 30 and 90 days.

### 100 patients records test

The chart review performed by a medical expert showed that 34 out of 100 randomly selected patients would be eligible for the NCT01018173-Roche study at UKM. The EHR4CR PF system identified eleven out of the 34 eligible patients (without false-positives). We found out that the medical expert had access to data from several clinics at the hospital rather than only from the clinic evaluated. To close this gap, all data available related to the given patient set derived from the seven clinics we had approvals from were loaded in the CDW for the given patient set. The EHR4CR PF system was then utilized again, which led to an increase of eligible patients found by the system: 23 out of 34 eligible patients with none false-positives. The remaining eleven mismatches were evaluated individually by accessing the respective patient records and discovering that the data related to the criteria which caused the EHR4CR PF system mismatches were solely written as free text in some of the clinical letters.

## Discussion

### Answers to study questions

This evaluation shows a relatively low accuracy in the counts obtained following both manual and EHR4CR PF supported processes at the two evaluated sites. The accuracy of the EHR4CR PF system depends on the quality of the data in the CDWs. A high level of quality that produces accurate results is only reached if the data in the EHR source is well documented and structured. If this is not the case, the EC become too restrictive and the EHR4CR PF system returns no matching patients. This can be stated after reviewing Tables 6 and 7: While the results in a less structured EHR as the one in UKM show an accuracy higher than PI responses in one out of eight cases and equal in other two (both counts are within the bounds of the gold standard), the results from APHP show a better accuracy of the EHR4CR PF system in two out of three cases. The importance of structure data is present in other studies [20] and clinicians should be made aware of it.

The process of obtaining patient counts using the EHR4CR PF system has proven not only to be faster than the current manual process (saving approximately seven calendar days per query) but also simpler and therefore less resource consuming. Once the EHR4CR PF system is set up, a user can query several sites without involving personnel from the clinics in the process.

Relevant differences have been discovered between the results obtained in the two different sites evaluated. One of them is based on an EHR with more structured data and the counts obtained using the EHR4CR PF system were much closer to the “alloyed” gold standard than the second site. The accuracy of the EHR4CR system varies depending on the kind and number of EC, becoming less accurate with a higher number or more exclusive EC. With the manual review of clinical records, the existence of matching patients for the trials in the EHRs was proved.

We have identified several reasons for the EHR4CR PF mismatches:

The use of free text as unstructured data was the principal reason for mismatches between the EHR4CR PF system and the “alloyed” gold standard. As an example, medication is often stored as free text. It was also discovered that the diagnosis, assumed to be always documented in a structured way, is often only available in the EHRs as free text. For the reviewed cases, this happened only when the diagnosis was not the primary diagnosis.

Some of the EC evaluated such as the FISH test or the biPTH lab value are just not part of any tests followed in the evaluated sites. Instead, there are similar test with different names. The solution would be to adapt the EC to the countries or sites evaluated.

The EHR4CR consortium uses several controlled terminologies and classifications such as SNOMED-CT and ICD-10, but the consortium has also created its own terminology. This terminology responds to the need of the study sponsors to query for EC not available or not found in the existing terminologies and classifications (e.g.: “Transplant list” if the patient is in a transplant list). The EHR4CR terminology has some limitations though: it is not controlled and quite complicated to map to the existing standards. The authors recommend that the elements belonging to this terminology should be either replaced by similar elements in the existing controlled terminologies and classifications or included as part of the EHRs in order to facilitate the ETL.

Another reason for the mismatches is that some of the data are just not electronically documented and/or could not be found, as for example data existent only on paper. This concurs with Kopcke’s findings [21], who discovered a significant lack of structured data to support clinical trials in EHRs.

In some cases, the data available in the EHRs were sufficient to deduce some of the criteria and therefore the eligibility of the patient. For example, the Hoehn and Yahr classification is not documented as such, but a score could be deduced from the physician notes. Currently this is only possible with a chart review and not computed by the current ETL.

These reasons are similar to the ones found by Hersh [22], who also provides possible solutions to them. Several studies have assessed the quality of data in EHRs and provide guidelines on how to improve it [23-25]. A site willing to become an EHR4CR data provider, should use these data quality guidelines in order to become a useful member of the project. Besides, the data elements in the EHR4CR terminology services need to be compliant with the data existing at the sites and the ETL processes improved in order to deal with the currently ignored data.

The responses from the physicians to the question: “What process do you usually follow to answer a potential sponsor’s feasibility/site questionnaire?” showed that the process followed in order to answer these questionnaires is based on the clinicians own experience and they normally do not check historical records for their responses. The EHR4CR PF system on the other hand, provides objective data based on historical data.

One of the biggest advantages of the EHR4CR PF system is its flexibility. In this context, flexibility can be defined as the ability to easily modify the EC and re-run queries using the platform. In the current process this would require the creation of a new questionnaire and re-contacting the clinicians involved in order to get the number of matching patients for the modified criteria. This study showed that it can take approximately seven days to obtain responses from clinicians whereas the EHR4CR system only needs a few minutes. Thus we can state that for every phase of modifications of criteria using the EHR4CR can save up to seven calendar days.

### Strengths and weaknesses of the study

The evaluation at two of the EHR4CR project sites included 900 manually checked patient records, 13 questionnaires answered by different clinicians and nine different EHR4CR databases with more than 30 thousand patients from seven different clinics. The participation of two sites from two different countries led to important findings and differences between them.

The simplification process of the study criteria might have caused mistakes in the final feasibility EC utilized. The root cause for this problem is that current EC are available as non-computable free text. In principle, this should not alter the evaluation as the same final EC were evaluated following the three methods (the simulation of the current process, the process using the EHR4CR PF system and the chart review). However, in the authors’ opinion, the EC used for some of the studies (e.g.: GSK – NCT00894387 with 26 EC before and after the simplification) could be closer to the final study criteria rather than just feasibility criteria, as they are normally simpler and less restrictive.

The feasibility questionnaires were only sent by EFPIA representatives in three of the cases, in the other ten cases the site had to approach the clinicians. The lack of commercial interest for this task might result in a delay on the response. This lack of interest is evident when the physicians where approached by the sites, resulting in some of the cases in a delay of more than three months. As this value is not realistic, the only measurement considered valid in a real-world setting was the one obtained when the site was approached by the EFPIA partner (seven calendar days).

Due to the shifted dates of the data in the CDWs, temporal constraints of the EHR4CR queries were adapted in order to obtain all possible true positive eligible patients, resulting in an eventual increase of false positives. However, the manual check of a representative set of patients for the study NCT01018173 suggests that EHR4CR queries tend to discover too few true positives, but no false positives.

### Results in relation to other studies

Despite the rising awareness of the importance of health information system evaluations, the authors could not find a similar evaluation to this one in the reviewed literature. There are several patient cohort systems being developed across the world. Some of the most prominent ones, such as the Shared Health Research Information Network (SHRINE) [26], the Feasibility Assessment and Recruitment System for Improving Trial Efficiency (FARSITE) [27] or the Electronic Primary Care Research Network (ePCRN) [28], also include the possibility to formulate complex queries and find eligible patients for a trial. These systems have in common that they operate with a single EHR data source. The EHR4CR PF system operates with several different data sources (MySQL, SQL Server, Oracle and PostgreSQL) and technologies. Moreover, these systems offer a quite limited capability in regard to their temporal reasoning when this is compared to the EHR4CR PF services [15].

### Meaning and generalizability of the study

This research has found crucial issues that hamper the re-use of EHR data for clinical research and proposes solutions for them. Besides, the study establishes a method to evaluate the efficacy and the effectiveness of similar systems. The same methodology or an adapted one could be re-used to assess the availability and quality of EHR data in a specific clinical site and its potential to become an EHR4CR data provider.

### Unanswered and new questions

The improvement of the ETL process must continue in order to reuse all available information within the EHR data sources. All of the reasons for the EHR4CR PF system mismatches need to be independently treated and the recommendations and guidelines for data quality previously cited in this manuscript considered as a solution for them. One of the most relevant improvements could be the inclusion of natural language processing tools in order to extract and transform the data stored in free text. Once these improvements have been adopted, a new evaluation should be performed to assess the EHR4CR’s ability to extract eligible patients. An alternative to the CI-Wilson score to build the gold standard in a future evaluation would be the mark and recapture methodology [29], although methods for diagnostic tests can only be applied if the data compared are binomial, i.e. if the gold standard is available for every observation. With the complete set of patient records manually checked and the patient list obtained using the EHR4CR system, a contingency table could be built and sensitivity and specificity ratios calculated.

## Conclusions

This study has demonstrated the potential improvement of the study design step of clinical trials with the use of a patient count cohort system. Such a system is able to provide patient counts in a short period of time, which reduces the workload on personnel from both the clinics and the study sponsors and accelerates the overall study design process. However, the system depends on the availability of structured data within the EHR data sources. When such data are available, objective and accurate patient counts can be obtained. At the current stage, EHRs need to be enhanced with better structured data to be easily re-used for research purposes.

## Additional files

Additional file 1:
**Feasibility questionnaire template.**
Additional file 2:
**Queries in ECLECTIC.**
Additional file 3:
**Clinician responses.**

## References

[1] Bollyky TJ, Cockburn IM, Berndt E (2010). Bridging the gap: improving clinical development and the regulatory pathways for health products for neglected diseases. Clin Trials Lond Engl.

[2] Dugas M, Amler S, Lange M, Gerss J, Breil B, Köpcke W (2009). Estimation of patient accrual rates in clinical trials based on routine data from hospital information systems. Methods Inf Med.

[3] Arain M, Campbell MJ, Cooper CL, Lancaster GA (2010). What is a pilot or feasibility study? A review of current practice and editorial policy. BMC Med Res Methodol.

[4] Getz K (2014). Improving protocol design feasibility to drive drug development economics and performance. Int J Environ Res Public Health.

[5] Soto-Rey I, Trinczek B, Karakoyun T, Dugas M, Fritz F (2014). Protocol feasibility workflow using an automated multi-country patient cohort system. Stud Health Technol Inform.

[6] Getz KA, Wenger J, Campo RA, Seguine ES, Kaitin KI (2008). Assessing the impact of protocol design changes on clinical trial performance. Am J Ther.

[7] Rajadhyaksha V (2010). Conducting feasibilities in clinical trials: an investment to ensure a good study. Perspect Clin Res.

[8] Kalra D, Schmidt A, Potts HWW, Dupont D, Sundgren M, De M (2011). R4CR Research Consortium: Case report from the EHR4CR project—A European survey on electronic health records systems for clinical research. IHealth Connec.

[9] Thakkar M, Davis DC (2005). Risks, barriers, and benefits of EHR systems: a comparative study based on size of hospital. Perspect Health Inf Manag AHIMA Am Health Inf Manag Assoc.

[10] Powell J, Buchan I (2005). Electronic Health Records Should Support Clinical Research. J Med Internet Res.

[11] Coorevits P, Sundgren M, Klein GO, Bahr A, Claerhout B, Daniel C (2013). Electronic health records: new opportunities for clinical research. J Intern Med.

[12] Home | IMI - Innovative Medicines Initiative. [http://www.imi.europa.eu/].

[13] EHR4CR: Electronic Health Records for Clinical Research. [http://www.ehr4cr.eu/].

[14] ISO/TC 159/SC 4: Ergonomic requirements for office work with visual display terminals (VDTs) — Part 11: Guidance on usability. ISO 9241–11:1998 1998.

[15] Bache R, Miles S, Taweel A (2013). An adaptable architecture for patient cohort identification from diverse data sources. J Am Med Inform Assoc.

[16] Ouagne D, Hussain S, Sadou E, Jaulent M-C, Daniel C (2012). The Electronic healthcare record for clinical research (EHR4CR) information model and terminology. Stud Health Technol Inform.

[17] Claassen JAHR (2005). The gold standard: not a golden standard. BMJ.

[18] Spiegelman D, Schneeweiss S, McDermott A (1997). Measurement error correction for logistic regression models with an “Alloyed Gold Standard.”. Am J Epidemiol.

[19] Wilson EB (1927). Probable inference, the law of succession, and statistical inference. J Am Stat Assoc.

[20] Hoyt RE, Yoshihashi A, Bailey NJ. Health Informatics: Practical Guide for Healthcare and Information Technology Professionals. [Raleigh, N.C.]: Lulu.com; 2012.

[21] Köpcke F, Trinczek B, Majeed RW, Schreiweis B, Wenk J, Leusch T (2013). Evaluation of data completeness in the electronic health record for the purpose of patient recruitment into clinical trials: a retrospective analysis of element presence. BMC Med Inform Decis Mak.

[22] Hersh WR, Weiner MG, Embi PJ, Logan JR, Payne PRO, Bernstam EV (2013). Caveats for the Use of Operational Electronic Health Record Data in Comparative Effectiveness Research: Med Care.

[23] Botsis T, Hartvigsen G, Chen F, Weng C (2010). Secondary use of EHR: data quality issues and informatics opportunities. AMIA Summits Transl Sci Proc.

[24] Institute of Medicine (US) Roundtable on Research and Development of Drugs, Biologics, and Medical Devices: Assuring Data Quality and Validity in Clinical Trials for Regulatory Decision Making: Workshop Report. Washington (DC): National Academies Press (US); 1999. 25077226

[25] Clark JS, Delgado VA, Demorsky S, Dunagan EA, Eichelmann TA, Hooper LA (2013). Assessing and improving EHR data quality (updated). J AHIMA Am Health Inf Manag Assoc.

[26] Weber GM, Murphy SN, McMurry AJ, Macfadden D, Nigrin DJ, Churchill S (2009). the shared health research information network (SHRINE): a prototype federated query tool for clinical data repositories. J Am Med Inform Assoc JAMIA.

[27] Ainsworth J, Buchan I (2009). Preserving consent-for-consent with feasibility-assessment and recruitment in clinical studies: FARSITE architecture. Stud Health Technol Inform.

[28] Peterson KA, Fontaine P, Speedie S (2006). The electronic primary care research network (ePCRN): a new era in practice-based research. J Am Board Fam Med JABFM.

[29] Chao A, Tsay PK, Lin SH, Shau WY, Chao DY (2001). The applications of capture-recapture models to epidemiological data. Stat Med.

