# Supplementary material for: Efficiency and effectiveness evaluation of an automated multi-country patient count cohort system
Source: BMC Med Res Methodol. 2015 May 1;15:44. doi: 10.1186/s12874-015-0035-9 (PMC4423123; doi:10.1186/s12874-015-0035-9)
Supplement: Additional file 1: — Feasibility questionnaire template. [file 12874_2015_35_MOESM1_ESM.docx]

October 31, 2013

Dear :

Within the project “Electronic Health Records For Clinical Research” (EHR4CR, <http://www.ehr4cr.eu>), conducted by several pharmaceutical companies and academic sites/university hospitals, we develop an automated support system for clinical trials. The first of the scenarios (protocol feasibility) has been successfully implemented and we would like to evaluate the system’s performance.

The clinical trial XXX (NCT/EudraCT Number XXX) is part of the evaluation of the project’s system. Since we would like to check how close the system’s answer is to an expert’s opinion, we kindly ask you to answer the following questions (following the process and tools you normally use for this kind of task).

The trial’s cohort is defined by the following set of inclusion and exclusion criteria:

Inclusion

- …
- …
- …

Exclusion

- …
- …
- …

Based on trial’s description, we’d like to know:

1. How many patients that fit to this study do you receive at your department/clinic/hospital per year?

___________ patients that fit to the study visit my department/clinic/hospital per year.

1. What process you usually run to answer a potential sponsor’s feasibility/site questionnaire? Please describe the tasks, personnel, documents/devices and (an estimation of) time invested briefly.

____________________________________________________________________________________________________________________________________________________________________________________________________________________________________

1. What are the major issues or time wasters you currently experience when answering feasibility questionnaires?

____________________________________________________________________________________________________________________________________________________________________________________________________________________________________

Thank you very much for participating in this survey.

Sincerely,
