# Supplementary material for: Efficiency and effectiveness evaluation of an automated multi-country patient count cohort system
Source: BMC Med Res Methodol. 2015 May 1;15:44. doi: 10.1186/s12874-015-0035-9 (PMC4423123; doi:10.1186/s12874-015-0035-9)
Supplement: Additional file 2: — Queries in ECLECTIC. [file 12874_2015_35_MOESM2_ESM.docx]

## BAYER - NCT00439725

1 born() at least 18 year before now and

2 last diagnosis([ICD-10:I26,"Pulmonary embolism"], [ICD-10:I80.2,"Phlebitis and thrombophlebitis of other deep vessels of lower extremities"]) and

3 last medication([ATC:B01AA03,"Warfarin [atc:B01AA03]"],
       [ATC:B01AA07,"Acenocoumarol [atc:B01AA07]"],
       [ATC:B01AX06,"Rivaroxaban [atc:B01AX06]"])

## AMGEN - NCT00345839

1 last vitalsign([LOINC:29265-6,"Calcium corrig albumine [Moles/Volume] S rum/Plasma ; Num rique"]) in range(>=2.09) unit([ucum:mmol/L,"millimole per liter"]) and

2 last vitalsign([LOINC:50676-6,"CaxP Pnl SerPl-mCnc"]) in range(>=3.85)
       unit([ucum:mmol/L,"millimole per liter"]) and

3 last vitalsign([EHR4CR:47,"biPTH"]) in range(>=159.0)
       unit([ucum:pg/mL,"picogram per milliliter"]) and

4 not last medication([ATC:H05BX01,"Cinacalcet [atc:H05BX01]"]) at least 11 month at most 17 month before now

## MERCK - NCT00627640

1 born() at least 30 year at most 80 year before now and
2 last diagnosis([ICD-10:G20,"Parkinson s disease"]) and
3 last medication([ATC:N04BA01,"Levodopa [atc:N04BA01]"]) and
4 last diagnosis([EHR4CR:46,"Motor fluctuations"]) and
5 not last status([SNOMED Clinical Terms:273518000,"Hoehn and Yahr grades"]) in {[EHR4CR:15,"Wheelchair bound or bedridden unless aided"]}

## NOVARTIS - NCT00894387

1 born() at least 18 year before now and

2 last diagnosis([ICD-10:I50,"Heart failure"]) at most 2 year before now and

3 last vitalsign([SNOMED Clinical Terms:250908004,"Left ventricular ejection fraction"]) in range(<=40.0) unit([ucum:%,"percent"]) at most 24 month before now and

4 last vitalsign([SNOMED Clinical Terms:271649006,"Systolic blood pressure"]) in range(>=109.0)
       unit([ucum:mm[Hg],"millimeter Mercury column"]) at most 2 year before now and

5 not last medication([ATC:C01D,"VASODILATORS USED IN CARDIAC DISEASES [atc:C01D]"],
       [ATC:C01CE,"Phosphodiesterase inhibitors [atc:C01CE]"],
       [ATC:C01C,"CARDIAC STIMULANTS EXCL. CARDIAC GLYCOSIDES [atc:C01C]"],
       [ATC:C01CA,"Adrenergic and dopaminergic agents [atc:C01CA]"]) and

6 not last diagnosis([ICD-10:O90.3,"Cardiomyopathy in the puerperium"],
       [ICD-10:I27.9,"Pulmonary heart disease, unspecified"]) and either

7 not last diagnosis([ICD-10:I21,"Acute myocardial infarction"]) at most 24 month before now or

8 not last procedure([SNOMED Clinical Terms:415070008,"Percutaneous coronary intervention"],
       [SNOMED Clinical Terms:64915003,"Operation on heart"]) at most 24 month before now and

9 not last procedure([SNOMED Clinical Terms:32413006,"Transplantation of heart"],
       [EHR4CR:6,"transplant list"]) and

10 not last diagnosis([ICD-10:I20.0,"Unstable angina"]) and

11 not last diagnosis([ICD-10:I27.9,"Pulmonary heart disease, unspecified"])

## JANSSEN - NCT00638690

1 born() at least 18 year before now and

2 last diagnosis([ICD-10:C61,"Malignant neoplasm of prostate"]) and

3 last procedure([SNOMED Clinical Terms:367336001,"Chemotherapy"]) and

4 last medication([ATC:L02BB03,"Bicalutamide [atc:L02BB03]"],

[ATC:L02BB01,"Flutamide [atc:L02BB01]"],

[ATC:L02BX03,"abiraterone"]) and

5 last vitalsign([LOINC:2857-1,"Prostate specific Antigen:Mass Concentration:Point in time:Serum/Plasma:Quantitative"]) in range(>=5.0)

unit([ucum:ng/mL,"nanogram per milliliter"]) and

6 not last medication([ATC:J02AB02,"Ketoconazole [atc:J02AB02]"])

## ASTRAZENECA - NCT00626548

1 gender() in {[SNOMED Clinical Terms:248153007,"Male"]} and
2 born() at least 18 year before now and
3 last diagnosis([ICD-10:C61,"Malignant neoplasm of prostate"])

## SANOFI - NCT00715624

1 last diagnosis([ICD-10:E11,"Non-insulin-dependent diabetes mellitus"]) and
2 last medication([ATC:A10AE,"Insulins and analogues for injection, long-acting [atc:A10AE]"],
       [ATC:A10AC,"Insulins and analogues for injection, intermediate-acting [atc:A10AC]"],
       [ATC:A10AD,"Insulins and analogues for injection, intermediate-acting combined with fast-acting"],
       [ATC:A10AB,"Insulins and analogues for injection, fast-acting"]) at most 24 month before now and
either
3 not last vitalsign([LOINC:4548-4,"Hemoglobin A1c/Hemoglobin.total:Mass Fraction:Point in time:Whole blood:Quantitative"]) in range(<=0.0)
       unit([ucum:%,"percent"]) or
4 not last vitalsign([LOINC:4548-4,"Hemoglobin A1c/Hemoglobin.total:Mass Fraction:Point in time:Whole blood:Quantitative"]) in range(>=0.0)
       unit([ucum:%,"percent"]) and
5 not last medication([ATC:A10BG03,"Pioglitazone [atc:A10BG03]"],
       [ATC:A10BG02,"Rosiglitazone [atc:A10BG02]"],
       [ATC:A10BG01,"Troglitazone [atc:A10BG01]"])

## ROCHE - NCT01018173

1 born() at least 18 year before now and
2 last diagnosis([ICD-10:E11,"Non-insulin-dependent diabetes mellitus"]) and
3 not last diagnosis([ICD-10:E10,"Insulin-dependent diabetes mellitus"])

## LILLY - NCT01468987

1 last diagnosis([ICD-10:E11,"Non-insulin-dependent diabetes mellitus"]) at most 24 month before now and
2 last vitalsign([SNOMED Clinical Terms:60621009,"Body mass index"]) in range(<=45.0)
       unit([ucum:kg/m2,"kilogram per square meter"]) and
3 not last medication([ATC:A10BX04,"Exenatide [atc:A10BX04]"],
       [ATC:A10BX07,"Liraglutide [atc:A10BX07]"],
       [ATC:A10BG,"Thiazolidinediones [atc:A10BG]"]) at most 24 month before now and
4 not last diagnosis([ICD-10:E16.2,"Hypoglycaemia, unspecified"],
       [ICD-10:E10.1,"Insulin-dependent diabetes mellitus with ketoacidosis"],
       [ICD-10:E10.0,"Insulin-dependent diabetes mellitus with coma"]) at most 24 month before now and
either
5 not last diagnosis([ICD-10:K71.7,"Toxic liver disease with fibrosis and cirrhosis of liver"],
       [ICD-10:K70.3,"Alcoholic cirrhosis of liver"]) or
6 not last vitalsign([LOINC:14631-6,"Bilirubin:Substance Concentration:Point in time:Serum/Plasma:Quantitative"]) in range(>=20.45)
       unit([ucum:umol/L,"micromole per liter"]) or
7 not last vitalsign([LOINC:1742-6,"Alanine aminotransferase:Catalytic Concentration:Point in time:Serum/Plasma:Quantitative"]) in range(>=124.0)
       unit([ucum:[iU]/L,"international unit per liter"]) or
8 not last vitalsign([LOINC:1920-8,"Aspartate aminotransferase:Catalytic Concentration:Point in time:Serum/Plasma:Quantitative"]) in range(>=125.0)
       unit([ucum:[iU]/L,"international unit per liter"]) and
9 not last diagnosis([ICD-10:C00-C97.9,"Malignant neoplasms"]) at most 6 year before now and
10 last medication([ATC:A10AE,"Insulins and analogues for injection, long-acting"],
       [ATC:A10AC,"Insulins and analogues for injection, intermediate-acting"],
       [ATC:A10AD,"Insulins and analogues for injection, intermediate-acting combined with fast-acting"],
       [ATC:A10AB,"Insulins and analogues for injection, fast-acting"])

## GSK

1 born() at least 18 year before now and
either
2 last status([SNOMED Clinical Terms:424122007,"ECOG performance status finding"]) in {[SNOMED Clinical Terms:425389002,"ECOG performance status - grade 0"]} or
3 last status([SNOMED Clinical Terms:424122007,"ECOG performance status finding"]) in {[SNOMED Clinical Terms:422512005,"ECOG performance status - grade 1"]} and
either
4 last status([Anatomic Pathology Lexicon (PathLex):430,"Breast-Infiltrating malignant neoplasm-pT"]) in {[Anatomic Pathology Lexicon (PathLex):717,"pT1c: Tumor &gt;10 mm but &lt; = 20 mm in greatest dimension"]} or
5 last status([Anatomic Pathology Lexicon (PathLex):430,"Breast-Infiltrating malignant neoplasm-pT"]) in {[Anatomic Pathology Lexicon (PathLex):718,"pT2: Tumor &gt;20 mm but &lt; = 50 mm in greatest dimension"]} or
6 last status([Anatomic Pathology Lexicon (PathLex):430,"Breast-Infiltrating malignant neoplasm-pT"]) in {[Anatomic Pathology Lexicon (PathLex):719,"pT3: Tumor &gt;50 mm in greatest dimension"]} or
7 last status([Anatomic Pathology Lexicon (PathLex):428,"Breast-Infiltrating malignant neoplasm-pN"]) in {[Anatomic Pathology Lexicon (PathLex):731,"pN1a: Metastases in 1 to 3 axillary lymph nodes, at least 1 metastasis greater than 2.0 mm"]} or
8 last status([Anatomic Pathology Lexicon (PathLex):428,"Breast-Infiltrating malignant neoplasm-pN"]) in {[Anatomic Pathology Lexicon (PathLex):730,"pN1mi: Micrometastases (greater than 0.2 mm and/or more than 200 cells, but none greater than 2.0 mm)."]} or
9 last status([Anatomic Pathology Lexicon (PathLex):428,"Breast-Infiltrating malignant neoplasm-pN"]) in {[Anatomic Pathology Lexicon (PathLex):732,"pN2a: Metastases in 4 to 9 axillary lymph nodes (at least 1 tumor deposit greater than 2.0 mm)"]} or
10 last status([Anatomic Pathology Lexicon (PathLex):428,"Breast-Infiltrating malignant neoplasm-pN"]) in {[Anatomic Pathology Lexicon (PathLex):733,"pN3a: Metastases in 10 or more axillary lymph nodes (at least 1 tumor deposit greater than 2.0 mm)"]} and
11 last procedure([SNOMED Clinical Terms:234262008,"Excision of axillary lymph node"]) and
12 last procedure([SNOMED Clinical Terms:367336001,"Chemotherapy"]) and
13 last vitalsign([SNOMED Clinical Terms:250908004,"Left ventricular ejection fraction"]) in range(>=49.0)
       unit([ucum:%,"percent"]) and
either
14 last status([Anatomic Pathology Lexicon (PathLex):433,"Breast-Infiltrating malignant neoplasm-HER2/neu (FISH method)"]) in {[Anatomic Pathology Lexicon (PathLex):741,"HER2/neu (FISH method)_Amplified (HER2 gene copy &gt;6.0 or ratio &gt;2.2)"]} or
15 last status([Anatomic Pathology Lexicon (PathLex):434,"Breast-Infiltrating malignant neoplasm-HER2/neu (immunoperoxidase study)"]) in {[Anatomic Pathology Lexicon (PathLex):1472,"HER2/neu (immunoperoxidase study)_Positive (Score 3+)"]} and
either
16 not last diagnosis([ICD-10:C50,"Malignant neoplasm of breast"]) at least 50 year before now or
17 not last medication([ATC:L01XC03,"Trastuzumab [atc:L01XC03]"]) at least 50 year before now and
either
18 not last status([Anatomic Pathology Lexicon (PathLex):430,"Breast-Infiltrating malignant neoplasm-pT"]) in {[Anatomic Pathology Lexicon (PathLex):720,"pT4: Tumor of any size with direct extension to the chest wall and/or to the skin (ulceration or skin nodules). Note: Invasion of the dermis alone does not qualify as pT4."]} or
19 not last status([Anatomic Pathology Lexicon (PathLex):430,"Breast-Infiltrating malignant neoplasm-pT"]) in {[Anatomic Pathology Lexicon (PathLex):721,"pT4a: Extension to chest wall, not including only pectoralis muscle adherence/invasion"]} or
20 not last status([Anatomic Pathology Lexicon (PathLex):430,"Breast-Infiltrating malignant neoplasm-pT"]) in {[Anatomic Pathology Lexicon (PathLex):722,"pT4b: Ulceration and/or ipsilateral satellite nodules and/or edema (including peau d orange) of the skin which do not meet the criteria for inflammatory carcinoma"]} or
21 not last status([Anatomic Pathology Lexicon (PathLex):430,"Breast-Infiltrating malignant neoplasm-pT"]) in {[Anatomic Pathology Lexicon (PathLex):723,"pT4c: Both T4a and T4b"]} or
22 not last status([Anatomic Pathology Lexicon (PathLex):430,"Breast-Infiltrating malignant neoplasm-pT"]) in {[Anatomic Pathology Lexicon (PathLex):724,"pT4d: Inflammatory carcinoma"]} and
23 last diagnosis([Anatomic Pathology Lexicon (PathLex):688,"Invasive ductal carcinoma (no special type or not otherwise specified)"],
       [Anatomic Pathology Lexicon (PathLex):689,"Invasive lobular carcinoma"],
       [Anatomic Pathology Lexicon (PathLex):2305,"Invasive tubular carcinoma"],
       [Anatomic Pathology Lexicon (PathLex):2302,"Invasive medullary carcinoma"],
       [Anatomic Pathology Lexicon (PathLex):2301,"Invasive mucinous carcinoma"],
       [Anatomic Pathology Lexicon (PathLex):2303,"Invasive papillary carcinoma"],
       [Anatomic Pathology Lexicon (PathLex):2306,"Invasive cribriform carcinoma"]) and
either
24 not last procedure([SNOMED Clinical Terms:278257006,"Peripheral blood stem cell graft"],
       [SNOMED Clinical Terms:23719005,"Transplantation of bone marrow"]) or
25 not last medication([ATC:L01DB01,"Doxorubicin [atc:L01DB01]"],
       [ATC:L01DB03,"Epirubicin [atc:L01DB03]"]) and
26 not last procedure([SNOMED Clinical Terms:428624002,"Radiotherapy to chest wall"]) and
27 not last diagnosis([ICD-10:C00-C97.9,"Malignant neoplasms"])

### Results UKM(0)

0 patients match all criteria

540 patients match: born() at least 18 year before now

0 patients match: last status([SNOMED Clinical Terms:424122007,"ECOG performance status finding"]) in {[SNOMED Clinical Terms:425389002,"ECOG performance status - grade 0"]}

0 patients match: last status([SNOMED Clinical Terms:424122007,"ECOG performance status finding"]) in {[SNOMED Clinical Terms:422512005,"ECOG performance status - grade 1"]}

0 patients match: last status([Anatomic Pathology Lexicon (PathLex):430,"Breast-Infiltrating malignant neoplasm-pT"]) in {[Anatomic Pathology Lexicon (PathLex):717,"pT1c: Tumor &amp;amp;gt;10 mm but &amp;amp;lt; = 20 mm in greatest dimension"]}

0 patients match: last status([Anatomic Pathology Lexicon (PathLex):430,"Breast-Infiltrating malignant neoplasm-pT"]) in {[Anatomic Pathology Lexicon (PathLex):718,"pT2: Tumor &amp;amp;gt;20 mm but &amp;amp;lt; = 50 mm in greatest dimension"]}

0 patients match: last status([Anatomic Pathology Lexicon (PathLex):430,"Breast-Infiltrating malignant neoplasm-pT"]) in {[Anatomic Pathology Lexicon (PathLex):719,"pT3: Tumor &amp;amp;gt;50 mm in greatest dimension"]}

0 patients match: last status([Anatomic Pathology Lexicon (PathLex):428,"Breast-Infiltrating malignant neoplasm-pN"]) in {[Anatomic Pathology Lexicon (PathLex):731,"pN1a: Metastases in 1 to 3 axillary lymph nodes, at least 1 metastasis greater than 2.0 mm"]}

0 patients match: last status([Anatomic Pathology Lexicon (PathLex):428,"Breast-Infiltrating malignant neoplasm-pN"]) in {[Anatomic Pathology Lexicon (PathLex):730,"pN1mi: Micrometastases (greater than 0.2 mm and/or more than 200 cells, but none greater than 2.0 mm)."]}

0 patients match: last status([Anatomic Pathology Lexicon (PathLex):428,"Breast-Infiltrating malignant neoplasm-pN"]) in {[Anatomic Pathology Lexicon (PathLex):732,"pN2a: Metastases in 4 to 9 axillary lymph nodes (at least 1 tumor deposit greater than 2.0 mm)"]}

0 patients match: last status([Anatomic Pathology Lexicon (PathLex):428,"Breast-Infiltrating malignant neoplasm-pN"]) in {[Anatomic Pathology Lexicon (PathLex):733,"pN3a: Metastases in 10 or more axillary lymph nodes (at least 1 tumor deposit greater than 2.0 mm)"]}

70 patients match: last procedure([SNOMED Clinical Terms:234262008,"Excision of axillary lymph node"])

1 patients match: last procedure([SNOMED Clinical Terms:367336001,"Chemotherapy"])

0 patients match: last vitalsign([SNOMED Clinical Terms:250908004,"Left ventricular ejection fraction"]) in range(>=49.0) unit([ucum:%,"percent"])

0 patients match: last status([Anatomic Pathology Lexicon (PathLex):433,"Breast-Infiltrating malignant neoplasm-HER2/neu (FISH method)"]) in {[Anatomic Pathology Lexicon (PathLex):741,"HER2/neu (FISH method)_Amplified (HER2 gene copy &amp;amp;gt;6.0 or ratio &amp;amp;gt;2.2)"]}

0 patients match: last status([Anatomic Pathology Lexicon (PathLex):434,"Breast-Infiltrating malignant neoplasm-HER2/neu (immunoperoxidase study)"]) in {[Anatomic Pathology Lexicon (PathLex):1472,"HER2/neu (immunoperoxidase study)_Positive (Score 3+)"]}

545 patients match: not last diagnosis([ICD-10:C50,"Malignant neoplasm of breast"]) at least 50 year before now

545 patients match: not last medication([ATC:L01XC03,"Trastuzumab [atc:L01XC03]"]) at least 50 year before now

545 patients match: not last status([Anatomic Pathology Lexicon (PathLex):430,"Breast-Infiltrating malignant neoplasm-pT"]) in {[Anatomic Pathology Lexicon (PathLex):720,"pT4: Tumor of any size with direct extension to the chest wall and/or to the skin (ulceration or skin nodules). Note: Invasion of the dermis alone does not qualify as pT4."]}

545 patients match: not last status([Anatomic Pathology Lexicon (PathLex):430,"Breast-Infiltrating malignant neoplasm-pT"]) in {[Anatomic Pathology Lexicon (PathLex):721,"pT4a: Extension to chest wall, not including only pectoralis muscle adherence/invasion"]}

545 patients match: not last status([Anatomic Pathology Lexicon (PathLex):430,"Breast-Infiltrating malignant neoplasm-pT"]) in {[Anatomic Pathology Lexicon (PathLex):722,"pT4b: Ulceration and/or ipsilateral satellite nodules and/or edema (including peau d orange) of the skin which do not meet the criteria for inflammatory carcinoma"]}

545 patients match: not last status([Anatomic Pathology Lexicon (PathLex):430,"Breast-Infiltrating malignant neoplasm-pT"]) in {[Anatomic Pathology Lexicon (PathLex):723,"pT4c: Both T4a and T4b"]}

545 patients match: not last status([Anatomic Pathology Lexicon (PathLex):430,"Breast-Infiltrating malignant neoplasm-pT"]) in {[Anatomic Pathology Lexicon (PathLex):724,"pT4d: Inflammatory carcinoma"]}

545 patients match: not last status([Anatomic Pathology Lexicon (PathLex):1903,"Breast-Infiltrating malignant neoplasm-pM"]) in {[Anatomic Pathology Lexicon (PathLex):2194,"cM0(i+) - No clinical or radiographic evidence of distant metastasis, but deposits of molecularly or microscopically detected tumor cells in circulating blood, bone marrow, or other nonregional nodal tissue that are &lt; = 0.2 mm in a patient without symptoms or signs of metastasis"]}

545 patients match: not last status([Anatomic Pathology Lexicon (PathLex):1903,"Breast-Infiltrating malignant neoplasm-pM"]) in {[Anatomic Pathology Lexicon (PathLex):2195,"pM1 - Distant detectable metastasis as determined by classic clinical and radiographic means and/or histologically proven &gt;0.2 mm"]}

0 patients match: last diagnosis([Anatomic Pathology Lexicon (PathLex):688,"Invasive ductal carcinoma (no special type or not otherwise specified)"], [Anatomic Pathology Lexicon (PathLex):689,"Invasive lobular carcinoma"], [Anatomic Pathology Lexicon (PathLex):2305,"Invasive tubular carcinoma"], [Anatomic Pathology Lexicon (PathLex):2302,"Invasive medullary carcinoma"], [Anatomic Pathology Lexicon (PathLex):2301,"Invasive mucinous carcinoma"], [Anatomic Pathology Lexicon (PathLex):2303,"Invasive papillary carcinoma"], [Anatomic Pathology Lexicon (PathLex):2306,"Invasive cribriform carcinoma"])

545 patients match: not last procedure([SNOMED Clinical Terms:278257006,"Peripheral blood stem cell graft"], [SNOMED Clinical Terms:23719005,"Transplantation of bone marrow"])

545 patients match: not last medication([ATC:L01DB01,"Doxorubicin [atc:L01DB01]"], [ATC:L01DB03,"Epirubicin [atc:L01DB03]"])

412 patients match: not last diagnosis([ICD-10:C00-C97.9,"Malignant neoplasms"])
