# Supplementary material for: Efficiency and effectiveness evaluation of an automated multi-country patient count cohort system
Source: BMC Med Res Methodol. 2015 May 1;15:44. doi: 10.1186/s12874-015-0035-9 (PMC4423123; doi:10.1186/s12874-015-0035-9)
Supplement: Additional file 3: — Clinician responses. [file 12874_2015_35_MOESM3_ESM.docx]

# Clinician responses to the questions:

1. What process you usually run to answer a potential sponsor’s feasibility/site questionnaire? Please describe the tasks, personnel, documents/devices and (an estimation of) time invested briefly.
2. What are the major issues or time wasters you currently experience when answering feasibility questionnaires?

## Clinician 1

1. We generally “look back in our memory” plus we look carefully for 2 weeks at our hospitalized and out-patients
2. We are following thousands of patients, but we do not have right at hand all the characteristics. Our cohort is insufficiently characterized for every aspects, type of diabetes, metabolic control, comorbidities, etc.. We have all the characteristics for specific sub-cohorts, for instance gestational diabetes, rendering investigations of course much easier. In theory, for hospitalized patients, there is a data base. The informatics department is supposed to help us in the future for the recruitment of clinical studies

## Clinician 2

1. Check the admissions and clinic visits, review on electronic case records, contacting the treating physician and the patient.
2. Too many irrelevant questions about the site and our experience.

## Clinician 3

1. Estimation of eligible patients for a study by experience or chart review of potential patients.
2. Time wasting review of charts or letters to give a relative precise number of eligible patients.

## Clinician 4

1. Evaluation of medical and institutional feasibility and consideration of medical needs as well as ethical aspects. Calculation of numbers of eligible patients. Reading the protocol synopsis, signing + faxing confidentiality agreement form, answering numerous questions on checking if our institution can handle needs of trials.
2. These questionnaires are always said to be answered within 5 minutes but when being answered thorough, it always takes 30 to 60 minutes. Most of the times, it is the same questions that have to be answered over and over again.

## Clinician 5

1. -
2. Many patients would be seen in a special department within the clinic (and therefore not all the clinicians have contact with them).

## Clinician 6

1. Feasibility questionnaires are filled out by myself (10-20 minutes) estimating the number of patients fulfilling the inclusion criteria and the amount of time and personnel is required.
2. To give a reliable estimate of the number of patients who fit to the study.

## Clinician 7

1. No standard process, it depends on the sponsor’s requests. 5 to 10 minutes (max).
2. Detailed statistics about the patient cohort missing. Sponsors requests insufficient.
